# The status of neglected tropical diseases amidst COVID-19 in Africa: Current evidence and recommendations

**DOI:** 10.34172/hpp.2021.53

**Published:** 2021-12-19

**Authors:** Rafiat Tolulope Akinokun, Esther Bosede Ilesanmi, Yusuff Adebayo Adebisi, Oluwadamilare Akingbade

**Affiliations:** ^1^Faculty of Nursing Science, Ladoke Akintola University of Technology, Ogbomoso, Nigeria; ^2^Institute of Nursing Research, Nigeria; ^3^Faculty of Pharmacy, University of Ibadan, Nigeria; ^4^The Nethersole School of Nursing, Chinese University of Hong Kong, Shatin, Hong Kong

**Keywords:** Africa, COVID-19, Neglected tropical disease

## Abstract

Health care services and programs directed towards combating the neglected tropical diseases (NTDs) have been disrupted because of the impact of the coronavirus disease 2019 (COVID-19). The African continent because of its staggering health care system and poor economy disproportionately bears the burden of these diseases. While successes have been recorded in controlling and eliminating the NTDs, policymakers in Africa should consider the potential of the COVID-19 to dwindle these successes an issue of high priority. This commentary seeks to discuss the current status of NTDs in Africa and proffer recommendations to help combat these diseases at this period. It is worthy to say that similar dedication directed towards fighting the COVID-19 should also be deployed into eliminating other diseases like the NTDs which often, are neglected.

## Introduction

Neglected tropical diseases (NTDs) are manifold group of communicable diseases caused by parasites, fungi, bacteria and viruses that are primarily found in tropical and subtropical climates.^1^ Globally, over one billion vulnerable and marginalized communities are negatively impacted by the burden of NTDs, with more impact on resource-constrained countries.^2^ Africa contributed 39% to the 1.5 billion of the world population affected by these diseases^3^ which made it a recognized public health problem in the region. NTDs are also known to be common among poor population living in tropical regions and hard-to-reach communities like the slums, rural environments and war-affected zones.^4^ African countries such as Nigeria and Democratic Republic of Congo ranked the first and second country hosting the highest number of schistosomiasis, onchocerciasis, as well as lymphatic filariasis in Africa respectively.^5,6^

Despite the health promotion and prevention programs on NTDs in Africa such as the World Health Organization (WHO) African Region NTDs programme, Global Eradication Efforts for Guinea Worm Disease, United States Government Initiative to Address NTDs and other global NTDs programs; the NTDs still persist.^4^ These efforts are further complicated by the coronavirus disease 2019 (COVID-19) pandemic in Africa. Since the declaration of the COVID-19 outbreak as a Public Health Emergency of International Concern by the WHO and the continent’s first confirmed case in Egypt on February 14, 2020; Africa continues to record daily rise of confirmed cases.^7,8^ Currently, Africa is experiencing the third wave of the pandemic, which further poses pressure on the fragile health systems and increased workload on the already stretched health workforce.^7^ Therefore, healthcare services delivery towards NTDs is greatly disrupted with stalled initiatives and financial commitments towards prevention and eradication strategies.^9^

## Vulnerability of Africa to NTDs

Popularly referred to as disease of poverty, the NTDs affect the most impoverished and marginalized people.^10^ Today, a larger concentration of poor people reside in Africa with about 490 million people in extreme poverty especially among the rural communities and the disadvantaged urban dwellers.^11^ This implies that Africans live below the World Bank international poverty line that stood at generating at least $1.9 financial income per day.^12^ Sustaining NTDs control is associated with integration of quality water supply, basic sanitation and personal hygiene programs. However, the vulnerable segment of the population lack these crucial amenities including access to health services and good housing.^13^ Low-income countries experience at least five NTDs happening concurrently as stated by the global health department of Centers for Disease Control and Prevention (CDC). The high burdens of these diseases are the source of physical and cognitive impairment, increased morbidity and mortality among women and children, reduced productivity rate which promote extreme poverty cycle.^14^

As COVID-19 pandemic evolves, population health and socioeconomic status of individuals and African nations are adversely affected.^15^ Africa’s economy experienced decrease in economic growth by 2.1% in 2020 based on the negative impact of the pandemic, according to the African Development Bank estimation.^16^ Majority of rural dwellers and people living with NTDs engage in agriculture including farming, fishery and animal husbandy. These activities were interrupted by the widespread of COVID-19 virus and resulted in production shutdown and decreased financial buoyancy. Additionally, lack of timely access to quality healthcare services and increased out-of-pocket payment attributed to the prevalence of NTDs in Africa.^17^

COVID-19 outbreak stretched the capacity of the health systems in Africa, promoting inaccessibility to quality health services. Despite the implementation of health insurance scheme in some African countries like Nigeria, Ghana and Kenya; a good number of the population affected by the NTDs and people at-risk still wallow in abject poverty due to disease co-morbidities and out-of-pocket expenditures.^18^ Although NTDs have desolating effects on human health and socioeconomic development, COVID-19 further intensified the undesirable effects associated with the diseases on the impoverished communities.^19^

## COVID-19 and NTD programs

Following the emergence of the COVID-19 pandemic and the public health measures to curtail the pandemic, the WHO noted suspension of activities directed towards active case-finding, mass treatment of NTDs, and population-based surveys for NTDs.^20^ However, interim guidance for the implementation of these programs was developed because a total halt of these programs may dwindle the hard-won successes towards NTD elimination and control.^21^ Models such as the NTD modeling consortium provided quantitative information about the impact of the COVID-19 pandemic on NTD elimination including ways to minimize these impacts.^22^ The consortium showed that the impact of the pandemic can still be mitigated provided there is prompt action to reverse these impacts.^23^ For NTD programs to resume to full functionality, there will be a need to establish innovative methods on how interventions are planned and implemented to achieve maximum health impacts and to build programs back and better.^24^

## Global health diplomacy and the fight against NTDs

To eradicate and control the NTDs in highly endemic regions, achieving universal treatment coverage and health services play a crucial role.^25^ At the 74th World Health Assembly in 2021, WHO endorsed a new 9 year (2021-2030) road map for NTDs with the aim of strengthening programmatic response to NTDs and promoting sustainable health systems with the use of cross-sectoral, integrated interventions, smart investment and community engagement.^26^ Being at the forefront of eradicating NTDs for years, WHO in 2005, created a Department for Control of Neglected Tropical Disease (WHO/NTD) in Geneva led by late Director-General Dr JW Lee at a time when it was urgent to initiate health intervention for millions living with the diseases.^19^ The 2021-2030 roadmap supersedes the 2012 roadmap by identifying the gaps and appropriate actions needed to achieve the new targets. Also, it shifted focus from disease-specific interventions to an integrated approach.^27^

The London Declaration which followed the WHO 2020 roadmap in 2012 organized by a group of partners integrated collaboration and commitment towards achieving universal health access for 10 of the NTDs, promoting the active involvement of global health actors including the pharmaceutical industries, philanthropic foundations, multilateral organizations and the government of the endemic countries in the eradication and control of NTDs. Ever since, essential medications and logistics have been made available to ensure the hard-to-reach communities and affected countries have access to NTDs-related treatment.^28^ Despite these efforts, about 600 million Africans are still denied access to treatment.^3^

Over the years, CDC with other partner organizations ensured proper implementation of available health interventions towards the eradication of NTDs. In 2020, COVID-19 negatively impacted the decades of actions and commitments from the global health actors towards improving health of affected people and prevention of occurrences among at-risk population, as resources are diverted to COVID-19 disease management and vaccine production.^29^ Social determinants of health made impoverished communities more susceptible to COVID-19 and NTDs. However, CDC strategies to reinforce human power towards disease surveillance and management, provision of accurate information for infection control and hospital preparedness assessment, identification of at-risk population including those vulnerable to disease co-morbidity and vaccine access for those in low and middle income countries contributed to the minimization of the disease burden.^8^ More than ever, the fight against COVID-19 further reiterate the power of synergistic action, strong political will and integrated intervention approach in the control and elimination of NTDs.

Furthermore, African Union in its Agenda 2063 goal indicated commitment to promoting healthy and well-nourished Africa, which is free of all NTDs, so also the Sustainable Development Goal 3 target 3 which focuses on 90% reduction in number of people seeking NTDs-related health interventions.^30^ Even though the diseases are prioritized in different agendas and roadmaps, the advent of COVID-19 pandemic sidelined the interventions towards eliminating the identified NTDs.

## Opportunity for innovation

The 2021-2030 roadmap which is the second WHO blueprint for the control and elimination of NTDs succeeded the initial blueprint developed in 2012 with milestones established for 2020.^31^ Although, many successes were recorded in the 2012-2020 roadmap; for instance, the Global Program to Eliminate Lymphatic Filariasis (GPELF) is regarded as one of the most successful public health programs.^32^ However, not all the milestones were met and this had implications for improving interventions, program management and delivery, diagnostic methods, and adequate financing mechanisms for each disease.^31^ The new 2021-2030 WHO road map for NTD programs focused on three major action areas which are to accelerate programmatic action against NTDs; intensify cross-cutting approaches by integrating interventions for several NTDs into national health systems; change operating models and culture by increasing country ownership.^31^

In March 2020, the Africa Union Commission revised its continental framework for NTDs (2021-2030) intending to guide member states in fighting NTDs in the continent and called for improved efforts in eliminating and controlling NTDs in the wake of the new WHO road map.^33,34^ The framework recognized strategic approaches to the elimination of NTDs such as increase financing, community engagement and ownership, effective partnerships and collaboration, and research, development, and innovative technologies.^34^ Country ownership of health systems will be successful in Africa if there is private investment.^35^ However, no African country has achieved their pledged target of 15% allocation of their annual budget towards the fight of NTDs.^36^

With the disruptions in the health system and economies of African countries caused by the pandemic, effective budget allocation is now paramount more than ever if the elimination of NTDs is to be achieved. To achieve health systems strengthening, community health workers which are a critical part of the African health care delivery system should be trained to deliver community-oriented primary care for disease surveillance and testing such as that implemented in South Africa.^35,37^ Researchers in Sub-Saharan Africa have identified inadequate human capacity, complex logistical and financial systems and delays in ethical reviews as barriers to conducting clinical trials. It is therefore important that African countries create a more enabling environment for research and innovation.^38^

## Conclusion and Recommendation

Achieving the control and elimination of NTDs should be of high priority in Africa because of the health benefits that come with eliminating communicable diseases and the impact of its elimination on the socio-economic development of Africa. Now more than ever, strong and effective partnership and collaboration with donors and relevant international bodies are essential to achieve adequate financing and development of endemic countries from their political leaders to their communities.^33^ Studies have shown that COVID-19 will further enhance global inequity, particularly in low and middle-income countries. Therefore, NTD programs with recognized success over the past years should remain a priority on the health and development agenda in Africa because of their roles in promoting many of the Sustainable Development Goals.^39^ We have seen the resources deployed towards combating COVID-19, similar dedication should also be demonstrated in fighting the NTDs.

## Acknowledgements

The authors appreciate Mr Iyiola Oladunjoye and the reviewers for their insightful comments in improving the quality of the manuscript.

## Funding

The authors received no financial support for the research, authorship, and/or publication of this article.

## Competing interests

The authors declare that they have no known competing financial interests or personal relationships that could have appeared to influence the work reported in this paper.

## Ethical approval

Not applicable.

## Authors’ contributions

RTA conceptualized the manuscript, EBI and RTA collected the relevant information and wrote the first draft of the manuscript, YAA edited the manuscript, OA reviewed the final draft of the paper and provided critical comments before submission.
